# Draft Genome Sequence of the Iron-Oxidizing, Acidophilic, and Halotolerant “*Thiobacillus prosperus*” Type Strain DSM 5130

**DOI:** 10.1128/genomeA.01042-14

**Published:** 2014-10-23

**Authors:** Francisco J. Ossandon, Juan Pablo Cárdenas, Melissa Corbett, Raquel Quatrini, David S. Holmes, Elizabeth Watkin

**Affiliations:** a Fundación Ciência & Vida, Santiago, Chile; b Facultad de Ciencias Biologicas, Andres Bello University, Santiago, Chile; c School of Biomedical Sciences, Curtin University, Perth, Australia

## Abstract

“*Thiobacillus prosperus*” is a halotolerant mesophilic acidophile that gains energy through iron and sulfur oxidation. Its physiology is poorly understood. Here, we describe the principal genomic features of the type strain of *T. prosperus*, DSM 5130. This is the first public genome sequence of an acidophilic halotolerant bacterium.

## GENOME ANNOUNCEMENT

“*Thiobacillus prosperus*” is a Gram-negative, halotolerant, acidophilic, mesophilic, and chemolithoautotrophic bacterium capable of oxidizing both iron and sulfur compounds (1). It was originally isolated from a marine geothermal field in Italy, and the type strain (DSM 5130) is able to tolerate up to 0.6 M NaCl and requires a minimum of 0.04 M Cl^−^ for growth (1). Its name is provisional, although there is some evidence that puts this organism inside the *Gammaproteobacteria* class (2). This organism has an active role in biomining in some salt-rich systems (3, 4). This is the first public genome sequence of an acidophilic halotolerant bacterium.

The genome of strain DSM 5130 was sequenced using the Ion Torrent sequencing platform. The library was constructed with the Ion Xpress Plus gDNA fragment library preparation kit. Quality-filtered reads were assembled *de novo* using Newbler (version 2.6). The draft genome size is 3.32 Mb, with a median coverage depth of 8.5-fold and an average G+C content of 64.46%. It contains 46 large contigs (>1,000 bp), with an *N*_50_ of 275,906 reads, and 25 smaller contigs. Its genes were identified using an in-house annotation pipeline that includes the use of databases and tools, such as AlterORF (5), Swiss-Prot (6), CDD (7), TnpPred (8), and Aragorn (9). The draft genome annotation predicts 48 tRNA sequences, 1 rRNA operon, and 3,088 protein-coding genes, 20.5% of which have no Clusters of Orthologous Groups (COG) assignation.

It is known that *T. prosperus* is an iron oxidizer, using a suite of genes similar to those found in the *pet-rus* operons from *Acidithiobacillus ferrooxidans* (4). Genes from these operons were found in *T. prosperus* genome. It is also known that this organism is a sulfur oxidizer (1). The genome analysis detected genes for sulfur oxygenase reductase (SOR), sulfide:quinone oxidoreductase (SQR), and the subunits SoxABCXYZ of the sulfur oxidation system, which are involved in sulfur oxidation (10). The *T. prosperus* genome also contains a complete set of genes for the Calvin-Benson-Bassham CO_2_ fixation cycle, including those for carboxysomes. Compatible solute biosynthesis is known to be one of the main adaptations for life in a high-osmolarity environment (11). The genome of *T. prosperus* has genes potentially involved in the biosynthesis of ectoine (diaminobutyrate aminotransferase, diaminobutyrate acetyltransferase, and ectoine synthase), sucrose (sucrose synthase), and glycine betaine (betaine aldehyde dehydrogenase). *T. prosperus* also encodes ABC-transporter systems for ectoine and glycine betaine uptake. Genomic analysis also predicted a complete set of genes involved in flagellum formation and chemotaxis, in agreement with previous studies demonstrating that *T. prosperus* has a polar flagellum (1). Interestingly, *T. prosperus* is predicted to have the Nif complex, which is involved in nitrogen fixation (12).

### Nucleotide sequence accession numbers.

This whole-genome shotgun project has been deposited at DDBJ/EMBL/GenBank under the accession no. JQSG00000000. The version described in this paper is version JQSG01000000.
